# Frailty Status as a Predictor of Outcomes in Emergency Surgeries for Older Adults: A Systematic Review and Meta-Analysis

**DOI:** 10.7759/cureus.84160

**Published:** 2025-05-15

**Authors:** Svetlana Doris Brincat, Clifford Caruana, Rajarshi Mukherjee

**Affiliations:** 1 Department of Surgery, Mater Dei Hospital, Msida, MLT; 2 Department of Emergency General and Major Trauma Surgery, Aintree University Hospital, Liverpool, GBR

**Keywords:** acute abdominal surgery, complications, elderly patients, emergency surgery, frailty, mortality

## Abstract

The global aging population has brought increasing attention to frailty as a critical predictor of health outcomes. Defined by the British Geriatric Society as a state of diminished physiological reserve across multiple systems, frailty reflects a heightened vulnerability to adverse events. While the negative impact of frailty is well established in elective surgical settings, its influence on outcomes following emergency abdominal surgery remains less clear. This meta-analysis evaluates postoperative outcomes in frail versus non-frail elderly patients undergoing emergency abdominal surgery. A comprehensive search of eight electronic databases was conducted from inception to January 2024, with an additional search in June 2024. Eligible studies were selected based on predefined inclusion criteria. The primary outcome was postoperative mortality, with secondary outcomes, including complications, length of hospital stay, discharge destination, readmission, and reoperation rates. Data were synthesized using RevMan5 (Cochrane Collaboration, London, UK) and R (R Development Core Team, Vienna, Austria), applying both fixed and random-effects models. Risk of bias in individual studies was assessed using the Quality in Prognostic Studies (QUIPS) tool. Thirty-one studies involving 1,750,195 participants were included. Frail patients showed significantly increased 30-day (OR: 2.83, 95% CI: 2.45-3.27; p<0.00001) and 12-month (OR: 1.97, 95% CI: 1.32-2.93; p=0.0008) mortality. They also experienced higher overall morbidity, more severe complications (Clavien-Dindo ≥3: OR: 2.39, 95% CI: 1.82-3.13; p<0.00001), longer hospital stays (WMD: 3.74 days, 95% CI: 1.54-5.94; p=0.0008), and increased rates of readmission and reoperation (OR: 1.48, 95% CI: 1.25-1.75; p<0.00001). Discharge to rehabilitation or skilled nursing facilities was also more common among frail patients. These findings demonstrate that frailty significantly worsens postoperative outcomes in elderly patients undergoing emergency abdominal surgery. Further research is warranted to explore the integration of frailty assessment tools in emergency settings to support surgical decision-making for this vulnerable population.

## Introduction and background

Frailty, a complex and multifaceted syndrome often seen in older adults, is described by the British Geriatric Society (2017) as a "distinctive health state related to the ageing process in which multiple body systems gradually lose their in-built reserves" [1]. This gradual decline reduces strength, endurance, and physiological function, increasing susceptibility to stressors [2,3]. As the global population ages, frailty is emerging as a pressing health burden, with individuals aged 65 and older making up 10% of the world’s population in 2022; a proportion expected to reach 16% by 2050 [4]. This demographic shift emphasises the urgency of addressing frailty, particularly as nearly 20% of all surgical procedures are projected to involve patients aged 75 and older by 2030 [5].

Frailty is now widely understood as a multidimensional, dynamic condition arising from the natural aging process, reflecting reduced physiological resilience and increased vulnerability to stressors [6,7]. It is closely linked to resilience - the ability to recover from adverse events - with frail individuals experiencing prolonged recovery or decline [8]. Although over 50 frailty assessment tools exist, no single gold standard has been established [9,10]. The most commonly used models are the frailty phenotype and the frailty index. The frailty phenotype [7] defines frailty by the presence of at least three of five criteria: unintentional weight loss, exhaustion, weakness, slow gait speed, and low physical activity. The frailty index [11] quantifies frailty based on the proportion of accumulated health deficits, including comorbidities, impairments, and geriatric syndromes, producing a score between 0 and 1. Diagnosis of frailty involves applying these tools in clinical or research settings, either through physical performance measures (phenotype) or comprehensive assessment of deficits (index), allowing risk stratification and guiding care decisions.

Elderly patients generally experience worse perioperative outcomes than younger individuals, with advanced age linked to a two- to fourfold increase in postoperative complications and mortality [12]. However, research shows that age alone is only one aspect of the overall risk for surgical patients, as patient-specific and procedural complexities play crucial roles. Frailty, affecting 25-50% of elderly surgical patients, serves as a more accurate predictor of health outcomes than age itself, particularly in this demographic [13]. Procedural variables also matter: elective surgeries carry a 1% mortality and 10% morbidity rate, while emergency surgeries drastically elevate these risks, with a 30-day mortality rate of 10% and a morbidity rate of 40%, placing frail elderly patients at the highest peri-operative risk of poor post operative outcomes [14].

Although frailty assessment tools are widely available, many surgeons still tend to ‘eyeball’ their patients’ fitness for surgery, resulting in subjective and inconsistent opinions. This is even more so in emergency surgery, whereby, in contrast to elective surgery, patients present at inconvenient hours, frequently with little background information and minimal time for preoperative planning. This makes emergency general surgery in the elderly population a challenge to the surgeon responsible for their care. Nonetheless, the impact of frailty in the elderly on postoperative outcomes, especially in emergency settings, remains underexplored, highlighting the need for this meta-analysis to appraise current literature to add further insights into the outcomes of frail elderly patients undergoing emergency abdominal surgery. The aim of this meta-analysis is to critically appraise and systematically evaluate the postoperative outcomes experienced by frail elderly patients and compare these to non-frail elderly patients undergoing emergency abdominal surgery.

## Review

Method

Protocol Compilation and Registration

The protocol was compiled and registered in PROSPERO (CRD42024527911) [15]. The review was reported in accordance with the Cochrane Handbook for Systematic Reviews of Interventions [16] and Preferred Reporting Items for Systematic Reviews and Meta-Analyses Protocol (PRISMA-P) standards [17].

Search Strategy

A systematic search was conducted across eight electronic databases, covering studies published from their inception up to 31 January 2024. The databases searched included Cochrane Library, CINAHL, EMBASE, Google Scholar, MEDLINE, PubMed, SCOPUS, and Web of Science. The search was repeated in June 2024. The keywords and Medical Subject Headings (MeSH) terms used for the search strategy were expedit* OR emerg* OR urgent OR unplan* OR unschedul* AND - surg* OR laparotom* OR cholecystectom* OR colectom* OR hernia* OR adhesion OR incision OR drain* OR intestin* OR obstruction* OR ulcer* OR append* OR abdom* OR bowel* OR operat* AND frail*.

References of accepted articles were also manually screened for potentially relevant studies to ensure no additional publications were missed.

Inclusion and Exclusion Criteria

The selection criteria followed the PECO (Participants, Exposure, Comparison and Outcomes) framework [18], with participants including patients ≥65 years of age undergoing emergency abdominal surgery and exposure being frailty assessed by an explicitly described frailty assessment tool as compared to non-frailty. The primary outcome included mortality rate at 30-day, 90-day, 180-day, 12 months, 18 months, 19 months, and in-hospital mortality. The secondary outcomes included postoperative complications, length of hospital stay, discharge location, readmission, and reoperation.

Only full-text comparative studies that met the established inclusion criteria and reported at least the primary outcome were accepted. The search was not time-limited but restricted to articles in the English language. Studies were excluded if the data did not directly compare outcomes between frail and non-frail patients or if the primary outcome of interest was not reported. Studies were excluded if the study involved mixed populations of patients treated both conservatively and surgically, or if it included data on procedures other than those performed as emergency abdominal surgeries. Studies that assessed frailty based on a single laboratory or imaging test (e.g., sarcopenia or hypoalbuminemia) were excluded as these do not capture the multidimensional nature of frailty. Studies performed on animal models, conference abstracts, letters to the editors, or review articles were not included. The authors were reached out to provide any missing information. If they did not reply or provide the necessary data, the studies were excluded.

Study Selection and Data Extraction

The articles were selected after a thorough screening process assessing sequentially the title, abstract, and the full text article according to the inclusion and exclusion criteria using Covidence®. Duplicate studies were removed. This process was conducted independently by two members of the research team to promote reliability. Discrepancies were discussed until consensus was reached. A PRISMA flow chart [17] summarising the study selection was compiled. Data were extracted from the accepted articles.

Quality Assessment

The quality of the articles was assessed using the QUality In Prognosis Studies (QUIPS) tool, based on the recommendation of the Cochrane Prognosis Methods Group [19,20]. The overall risk of bias of individual studies was calculated following the study by Grooten et al. [21]. A study was classified as low risk if all domains were low risk or had up to one moderate risk. Studies with one or more high-risk domains or three or more moderate-risk domains were classified as high risk. All others were categorised as moderate risk.

Data Analysis

Data were analysed using RevMan Web. The difference in effect size was assessed using weighted mean differences for continuous variables by the application of the inverse variance method. For dichotomous variables, the Mantel-Haenszel method was used to calculate the odds ratio. For studies that provided the median, the standard deviation was calculated from the interquartile range. Statistical significance was set at p<0.05. Heterogeneity was assessed using the Cochrane Q test, I^2^, and Tau^2^, and this determined whether random-effect or fixed-effect models were applied. Considering potential heterogeneity among studies, the results were pooled using a random-effects model. If p>0.1 and I^2^<50%, heterogeneity was not considered significant, and the fixed effect model was used. Based on the Cochrane handbook, the degree of heterogeneity was classified as not important (0-40%), moderate (30-60%), substantial (50-90%), and considerable (75-100%) heterogeneity.

For studies reporting data using multiple frailty scores, we chose the data from the most commonly used frailty score for that specific outcome in our meta-analysis to enhance consistency and enable more reliable pooling of results.

Subgroup analysis was pre-defined and performed based on the different frailty scores. It was conducted for the main outcomes and when a sufficient number of studies were available. A p-value of less than 0.10 was only used to determine statistical significance in subgroup analysis [22].

Sensitivity analysis was then employed to assess the reliability of the results. Studies were limited to sample size (restricted to articles with 100 participants in each arm), study design (limited to prospective studies), quality (included studies with a low risk of bias only), and surgery type (restricted to studies that included laparotomies only).

Publication bias was assessed using funnel plots.

Results

Literature Selection and Description of Included Studies

**Figure 1:**
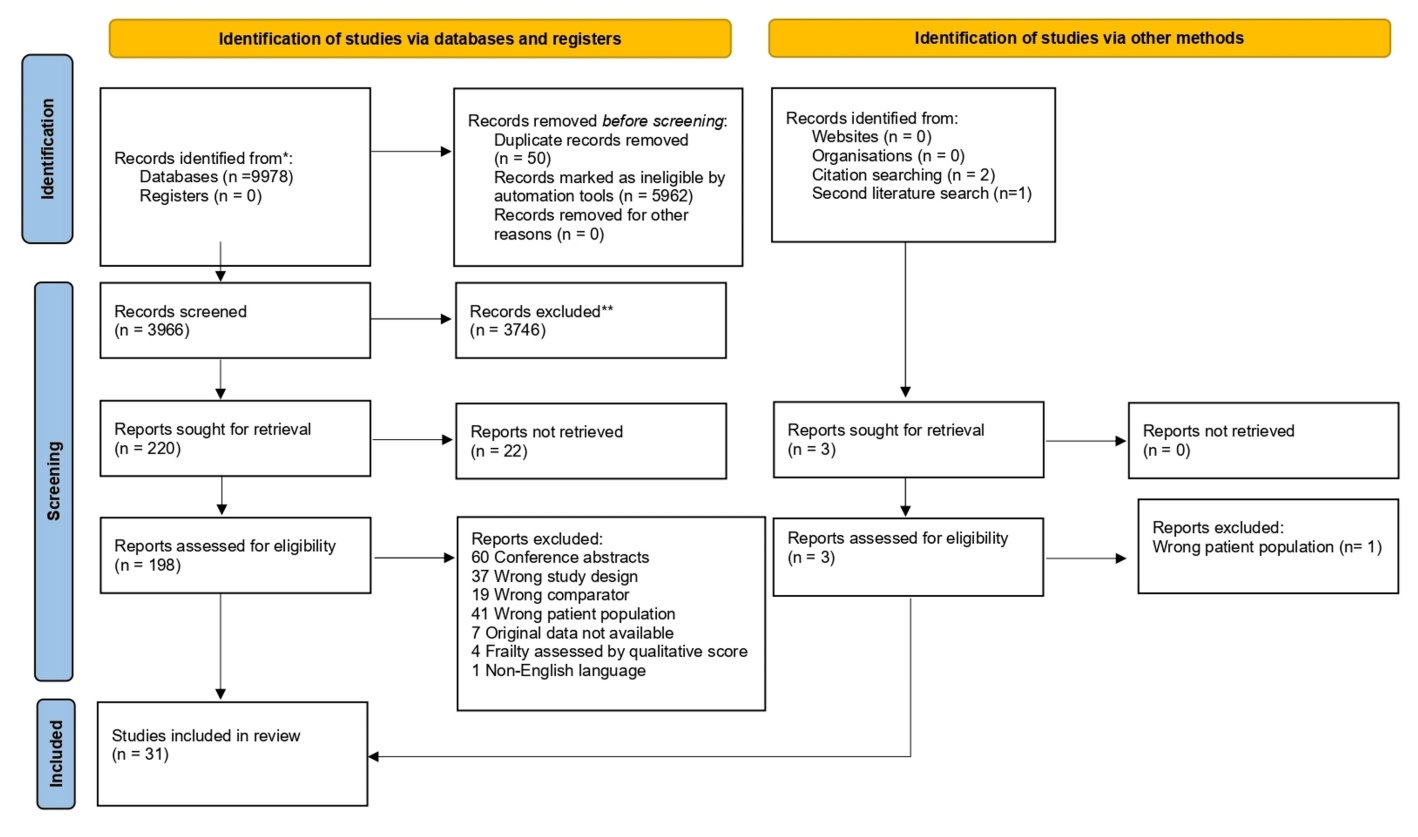
PRISMA flowchart PRISMA: Preferred Reporting Items for Systematic Reviews and Meta-Analyses

The combined literature search yielded 9,978 articles, as shown in the PRISMA flow diagram (Figure 1).

After removing duplicates, 3,966 studies were screened, with 220 full-text studies assessed for eligibility. The first literature search was conducted at the end of January 2024, whilst the second literature search was performed in early June 2024. A total of 31 studies met the inclusion criteria and were included in this meta-analysis [23-53].

Study and Participant Characteristics

The characteristics of the included studies are shown in Table 1.

**Table 1:** Basic study and participants’ characteristics

Study characteristics						Participants’ characteristics				
Author	Country	Centre/Database	Design	Study period	Frailty score	Total patients	Male/Female	Age (mean ± SD)	Frail (n)	Non-frail (n)
Alder et al. 2021 [23]	UK	Queen Alexandra Hospital	R	Jul 2015-Jul 2016	CFS	153	96/57	^†^79 (75–84)	74	79
Alkadri et al. 2022 [24]	USA	Health administrative data from the Canadian province of Ontario	R	Apr 2009-Mar2019	pFI	7003	3627/3376	77	2063	4940
Arteaga et al. 2021 [25]	Spain	Hospital Universitario Virgen del Rocío in Seville	P	Sep 2017-Apr 2019	CFS, FRAIL scale, TRST Share-FI	92	43/49	78.71 ± 6.26	23	69
Sánchez Arteaga et al. 2022 [26]	Spain	Hospital Universitario Virgen del Rocío in Seville	P	Sep 2017-Jun 2020	CFS, FRAIL scale, TRST	82	39/43	78.5	21	61
Castillo-Angeles et al. 2021 [27]	USA	Medicare inpatient claims file	R	Jan 2007-Dec 2015	CFI	882929	399292/483637	77.9 ± 7.5	11,1513	77,1416
Collins et al. 2023 [28]	USA	ACS NSQIP database	R	2012-2017	mFI-5	47216	20751/26465	72.9 ± 6.4	13,039	34,177
Costa et al. 2021 [29]	Italy	38 Italian centers	P	Jan 2017-Jun 2018	EmSFI	1024	574/454	77.82 ± 7.77	500	524
Hacim et al. 2021 [30]	Turkey	Bagcilar Training and Research Hospital in Istanbul	R	Feb 2016-Jan 2020	mFI-11	150	79/71	^†^74	24	126
Isand et al. 2023 [31]	UK	Single centre, Surgical Emergency Unit at the Oxford University Hospitals NHS Trust	R	Jan 2018-Jun 2021	CFS	411	194/217	76.9 ± 7.3	160	251
Orouji Jokar et al. 2016 [32]	USA	University Arizona College of Medicine; single centre	P	2013-2014	Modified Rockwood Frailty index; EGSFI	60	33/27	75.4 ± 7.8	18	42
Joseph et al. 2016 [33]	USA	University Arizona College of Medicine; single centre	P	Oct 2012-Mar 2014	Modified Rockwood frailty index	220	123/97	75.5 ± 7.7	82	138
Kapadia et al. 2022 [34]	USA	Division of Trauma, Critical Care, and Emergency Surgery, University of Arizona	P	2011-2017	EGSFI	458	339/119	74 ± 8	146	312
Kenawy et al. 2021 [35]	USA	ACS NSQIP database	R	2012-2017	mFI-5	47,216	20751/26465	75	13,039	34,177
Kenig et al. 2016 [36]	Poland	Tertiary referral hospital	P	Jun 2014-Dec 2015	GA – cumulative deficit model	60	26/34	^†^76	46	14
Kenig et al. 2018 [37]	Poland	Secondary referral hospital	P	Jan 2013-Dec 2016	G8	315	150/165	^†^77	190	125
Khan et al. 2019 [38]	USA	Level I trauma center -Banner University Medical Center, Tucson)	P	2014-2016	EGSFI	326	187/139	73.9 ± 8	127	199
Lee et al. 2020 [39]	USA	Database: Medicare claims	R	Jan 2008-Dec 2014	CFI	468459	196,557/271,902	79.5 ± 7.75	74,859	393,600
Li et al. 2018 [40]	USA	2 tertiary care hospitals in Canada (University of Alberta Hospital, Edmonton &Foothills Medical Centre Calgary)	P	Jan 2014–Sep 2015	CFS	308	168/140	^†^75	68	240
McGuckin et al. 2018 [41]	UK	University College Hospital, London	R	Jun 2012-Jan 2013	CFS	38	20/18	77.1 ± 8.3	11	27
McIsaac et al. 2017 [42]	Canada	Administrative data in Ontario	R	Apl 2002-Mar 2014	ACG (Johns Hopkins)	77,184	34961/42223	77.5 ± 7	19,779	57,405
Parmar et al. 2021 [43]	UK	Multicentre: 49 registered sites in the UK	P	Mar-Jun 2017	CFS	937	397/540	76 ± 6.82	190	747
Pigeon et al. 2023 [44]	Canada	Single-center	R	Jan 2016-Dec 2020	mFI-11	299	128/171	^†^82 (5)	163	136
Reinisch et al. 2022 [45]	Germany	Three participating centers	R	Jan 2015-Sep 2020	mFI-5	181	91/90	75.8 ± 7.5	19	162
Rosa et al. 2023 [46]	Italy	Single-center	R	Jan 2018-Sep 2021	CFS	358	190/168	^†^74	99	259
Salzman et al. 2022 [47]	USA	ACS NSQIP database	R	2016-2018	mFI-5	5728	2677/3051	^†^71 (67-76)	979	4749
Simon et al. 2020 [48]	USA	ACS-NSQIP database	R	2012-2016	mFI-5	10025	4195/5830	^†^75 (70–81)	3129	6896
Sokas et al.2021 [49]	USA	Master Beneficiary Summary File, denominator file and MedPAR files	R	Jan 2008-Dec 2014	CFI	138916	61,214/77,702	80.63 ± 7.8	34,892	104,024
Vilches-Moraga et al. 2020 [50]	UK	Salford Royal NHS Foundation Trust	P	Sep2014-Mar 2017	CFS	113	53/60	81.9 ± 4.7	37	76
Youseff et al. 2022 [51]	UK	United Lincolnshire Hospitals NHS Trust	R	Dec 2018-Mar 2021	CFS	191	106/85	^†^75	90	101
Zakhary et al. 2024 [52]	USA	ACS NSQIP database	R	2018-2020	mFI-5	59633	26,909/32,724	75	2549	57,084
Zattoni et al. 2019 [53]	Italy	Tertiary referral hospital	P	Dec 2015-May 2016	fTRST	110	47/63	81 (70–96)	72	38

The studies included in this review are contemporaneous and published from 2016 to 2024; 19 of the included studies were published in the past three years. Geographically, most studies were conducted in the USA (n=14), followed by the United Kingdom (n=6). All included studies were observational, with the majority of the studies (n=18) being retrospective in nature. In the included studies, various frailty assessment scores were utilised, with the CFS score being the most commonly used tool. Different studies employed varying cut-off criteria for frailty assessment. Most studies (n=23) included patients aged more than 65 years, followed by six studies which included participants aged more than 70 years.

In total, this meta-analysis encompasses data from 1,750,195 participants, of whom 278,001 were identified as frail. The frailty prevalence was 32.4%, ranging from 4.3% to 76.7%. The majority of the participants were females (n=976,182). The sample size ranged from 38 to 882,929 participants. The majority of emergency abdominal surgeries were laparotomies. The mean age of patients was 74.3 years.

Quality of Included Studies

**Figure 2:**
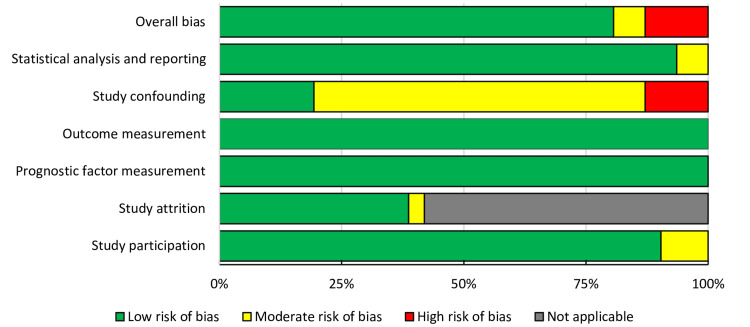
Risk of bias of studies using the QUIPS tool QUIPS: QUality In Prognosis Studies

The risk of bias of individual studies was evaluated using the QUIPS tool by two members of the research team (Figure S1). Overall, most studies were found to have a low (n=25) to moderate risk (n=2) of bias, with only four studies rated as high risk, primarily due to presence of confounding factors (Figure 2).

Primary Outcome: Mortality

**Figure 3:**
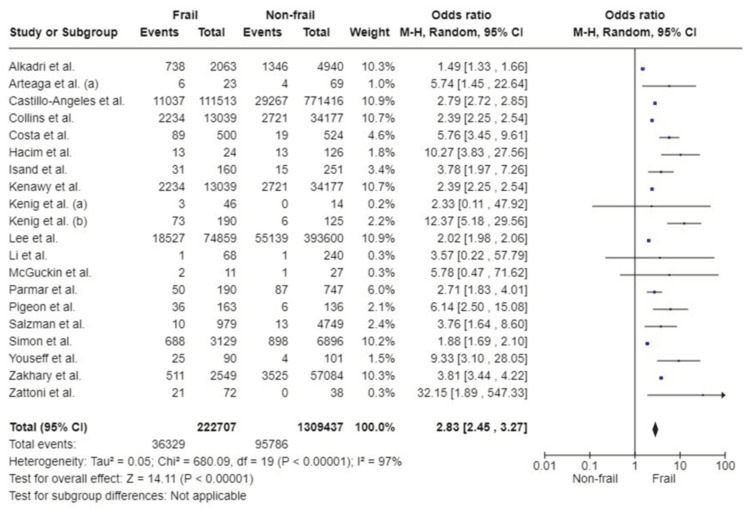
Forest plot of the association of frailty with 30-day mortality References: [24,25,27-31,35-37,39-41,43,44,47,48,51-53]

30-Day mortality: A total of 20 studies using various frailty assessment scores examined the association of frailty with 30-day mortality following emergency surgery. Analysis of the results included a total of 1,532,144 patients, with 222,707 patients in the frail group and 1,309,437 patients in the non-frail group. Pooled analysis showed that patients living with frailty had a statistically significant increase in mortality within 30 days postoperatively compared to the non-frail group (OR: 2.83; 95%CI: 2.45, 3.27; p<0.00001). Significant level of heterogeneity was detected (I^2^=97%, p<0.00001) (Figure 3).

**Figure 4:**
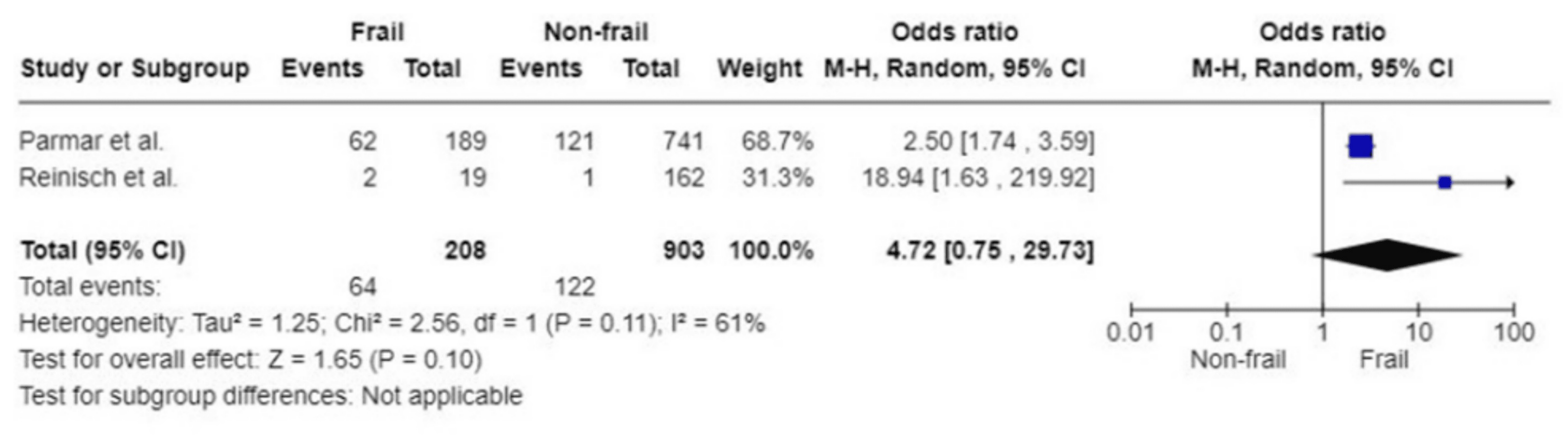
Forest plot of the association of frailty with 90-day mortality References: [43,45]

90-Day mortality: Only two studies, based on 208 participants in the frail group and 903 participants in the non-frail group, reported 90-day mortality postoperatively. Frail patients who underwent surgery have a fourfold likelihood of dying within 90 days postoperatively (OR: 4.72; 95%CI: 0.75, 29.73; p=0.10). Substantial heterogeneity amongst the studies was detected (I^2^=61%, p=0.11) (Figure 4).

**Figure 5:**
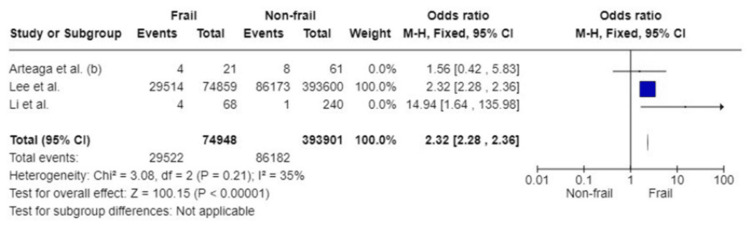
Forest plot of the association of frailty with 180-day mortality References: [25,39,40]

180-Day mortality: The association between frailty and the 180-day mortality rate was assessed in three studies. Pooled analysis based on 468,849 patients showed that patients with frailty have a twofold likelihood of mortality at 180 days postoperatively (OR: 2.32; 95%CI: 2.28, 2.36; p<0.00001). Moderate heterogeneity amongst studies was detected (I^2^=35%, p=0.21) (Figure 5).

**Figure 6:**
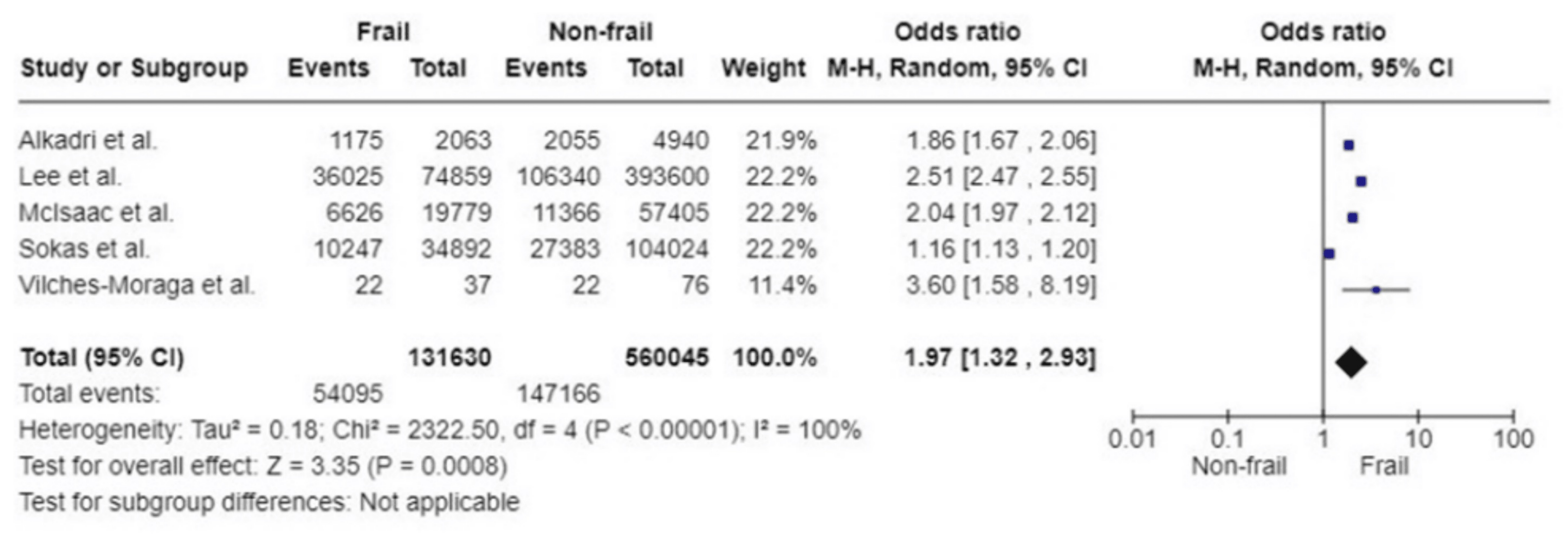
Forest plot of the association of frailty with 12-month mortality References: [24,40,42,49,50]

12-Month mortality: Five studies based on a total of 691,675 participants reported data on 12-month mortality following emergency abdominal surgery. Patients living with frailty had an almost two-fold increased risk of mortality 12 months postoperatively compared to their non-frail counterparts (OR: 1.97; 95%CI: 1.32, 2.93; p=0.0008). Caution needs to be exerted in interpreting this result as the studies included for this outcome assessed frailty by four different frailty assessment scores, and there is considerable heterogeneity amongst the included studies (I^2^=100%, p<0.00001) (Figure 6).

18-Month mortality: The study of Arteaga et al. [26] assessed 18-month mortality in patients aged >70 years undergoing emergency abdominal surgery. The surgeries performed were mainly laparotomies and inguinal hernia repairs. It showed that frail patients have a twofold higher mortality at 18 months after their emergency surgery (OR: 2.76; 95%CI: 1.02, 7.56; p=0.043).

19-Month mortality: Only the study of Alder et al. [23] assessed mortality outcomes at a median follow-up of 19 months postoperatively. Hence, pooled analysis for this outcome could not be generated. This study showed that frail patients who underwent emergency laparotomies have a three times higher risk of mortality at 19 months (OR: 3.2; 95%CI: 1.09, 9.61; p=0.034).

**Figure 7:**
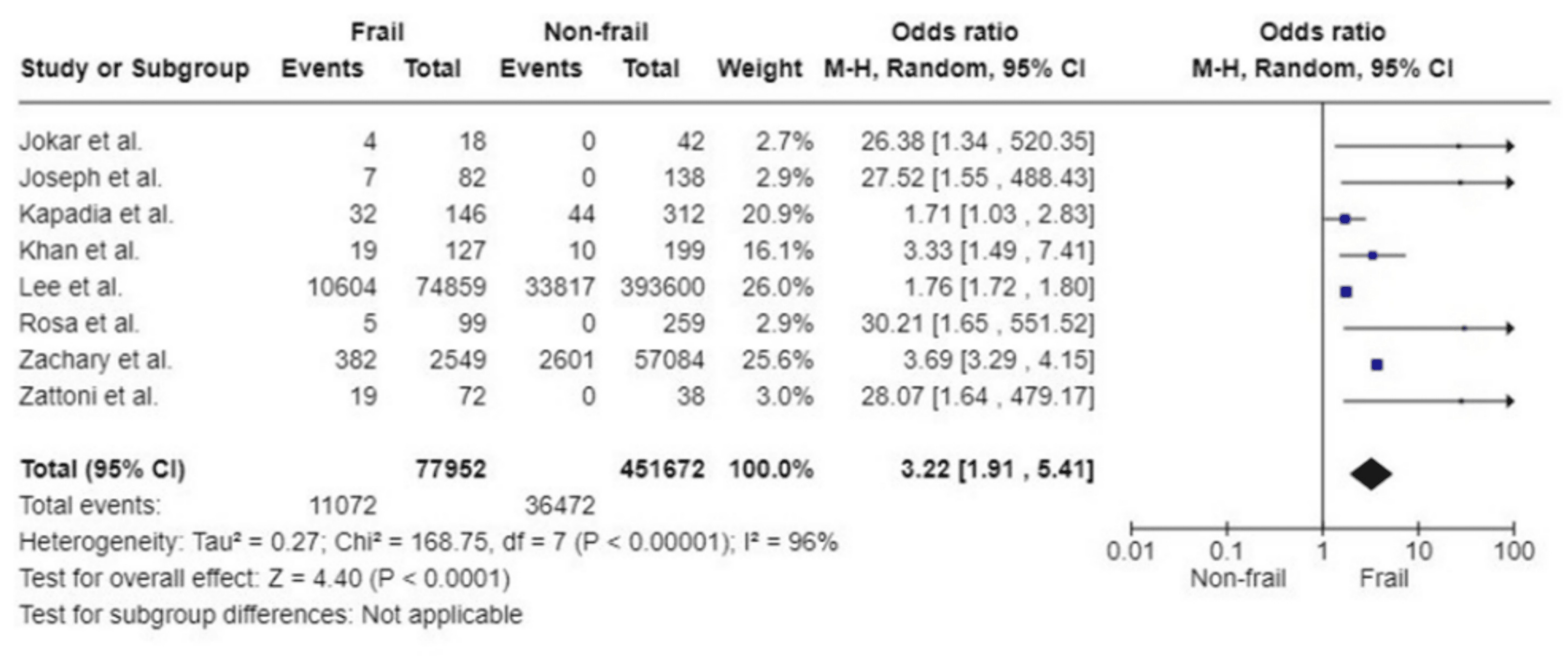
Forest plot of the association of frailty with in hospital mortality References: [32-34,38,39,46,52,53]

In-hospital mortality: The association between frailty and in-hospital mortality was assessed in eight studies covering 529,624 participants. Patients living with frailty had a threefold likelihood of mortality during their in-hospital stay postoperatively (OR: 3.22; 95%CI: 1.91, 5.41; p<0.0001). Considerable heterogeneity was present amongst the included studies (I^2^=96%, p<0.00001) (Figure 7).

Secondary Outcomes

**Figure 8:**
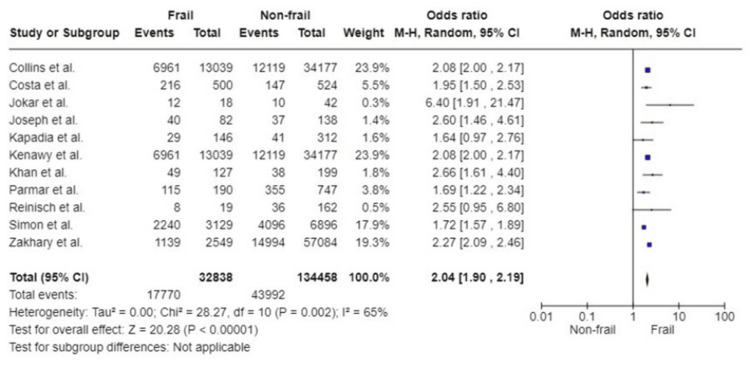
Forest plot of the association of frailty with postoperative complications References: [28,29,32-35,38,43,45,48,52]

Postoperative complications: Eleven studies reported the incidence of postoperative complications. The results were based on a total of 167,296 patients, with 32,838 participants considered as frail. All studies showed a greater risk of postoperative complications in patients who were assessed to be frail. Pooled analysis showed that patients living with frailty have a twofold increased likelihood of having complications postoperatively. This finding was statistically significant (OR: 2.04; 95%CI: 1.90, 2.19; p<0.00001). Substantial heterogeneity was detected (I^2^=65%; p=0.002) (Figure 8).

Severity of Complications

**Figure 9:**
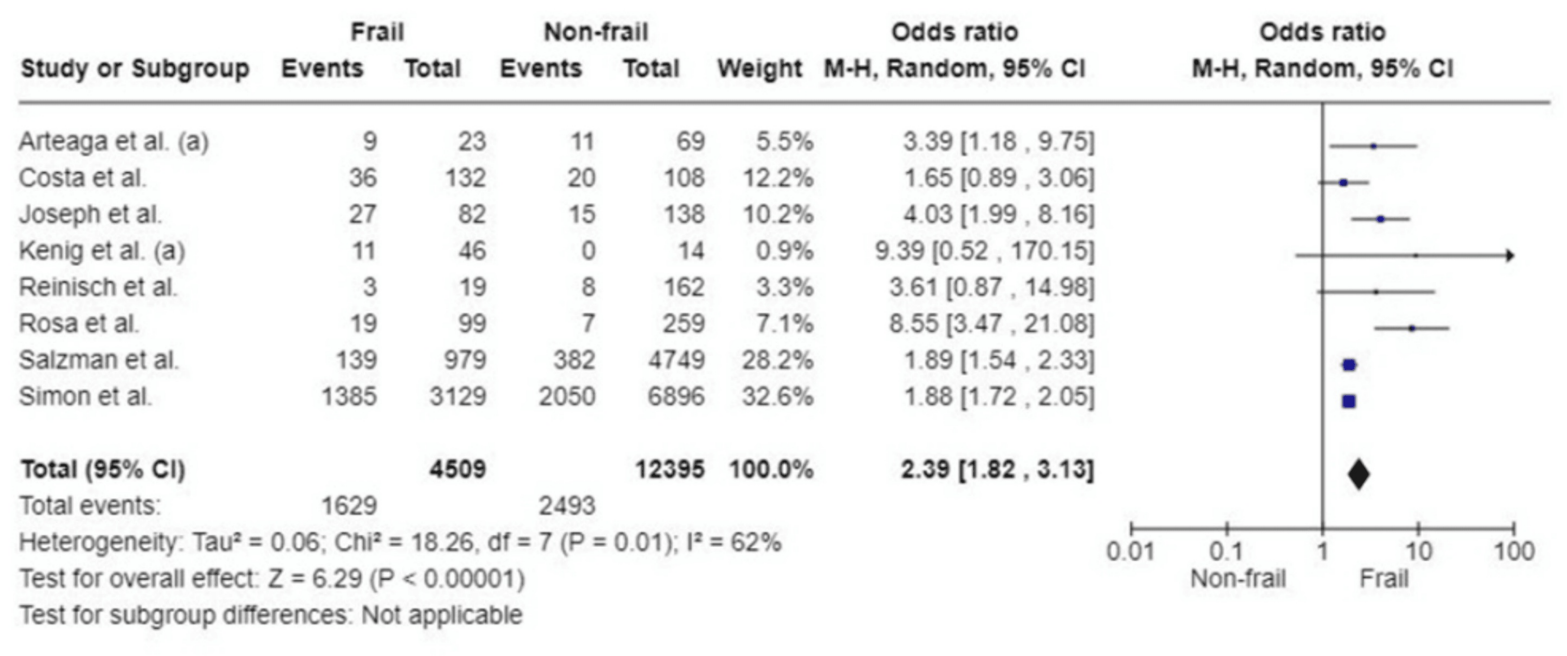
Forest plot of the association of frailty with severity of complications References: [25,29,33,36,45-48]

The Clavien-Dindo classification was utilised in studies to categorise the severity of complications. This is a well-established and validated grading system for surgical complications. Eight studies reported the association between frailty and severity of postoperative complications (classified as Clavien-Dindo grade≥III). Patients with frailty had a twofold increased risk of having serious complications. This outcome was found to be statistically significant (OR: 2.39; 95%CI: 1.82, 3.13; p<0.00001). Substantial heterogeneity amongst studies was detected (I^2^=62%, p=0.01) (Figure 9).

Intensive Care Unit (ICU) Admission

**Figure 10:**
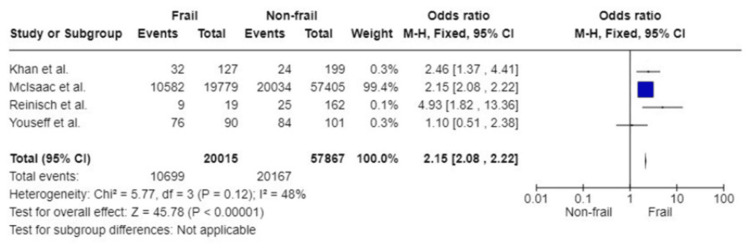
Forest plot of the association of frailty with ICU admission References: [38,42,45,51]

Four studies covering a total of 77,882 participants analysed frailty and ICU admission. Pooled analysis showed that the frail group had a statistically significantly higher likelihood of being admitted to the ICU following emergency surgery (OR: 2.15; 95%CI: 2.08, 2.22; p<0.0001). Moderate heterogeneity was detected in the study population (I^2^=48%, p=0.12) (Figure 10).

ICU Length of Stay

**Figure 11:**
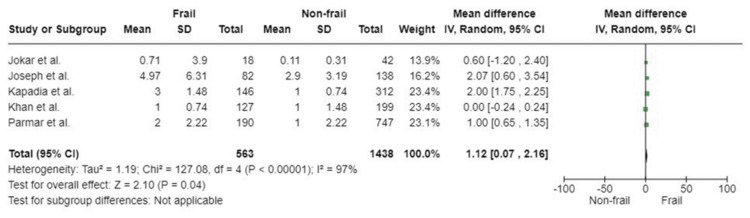
Forest plot of the association of frailty with ICU length of stay References: [32-34,38,43]

Five included studies, based on a total of 2,001 participants, investigated the association between frailty and ICU length of stay. The analysis showed a significant difference between the ICU length of stay of patients living with frailty and non-frail patients (WMD: 1.12; 95%CI: 0.07, 2.16; p=0.04). Substantial heterogeneity was detected in the included studies (I^2^=97%, p<0.00001) (Figure 11).

Failure to Rescue (FTR)

**Figure 12:**
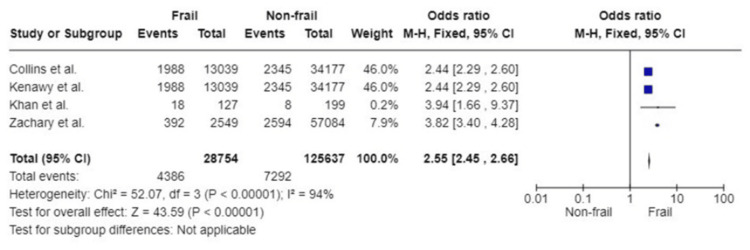
Forest plot of the association of frailty with failure to rescue References: [28,35,38,52]

FTR refers to the inability or delay in identifying and addressing complications (including mortality) experienced by a hospitalised patient due to a disease process or medical intervention [54]. Four studies with a total of 154,391 participants reported outcomes of frailty in terms of FTR. Results from the pooled data showed that frail patients had an increased likelihood of having FTR following emergency abdominal surgery compared to the non-frail participants (OR: 2.55; 95%CI: 2.45, 2.66; p<0.00001). Considerable heterogeneity was detected with an I^2^ of 94% (p<0.00001) (Figure 12).

Duration of Hospital Stay

**Figure 13:**
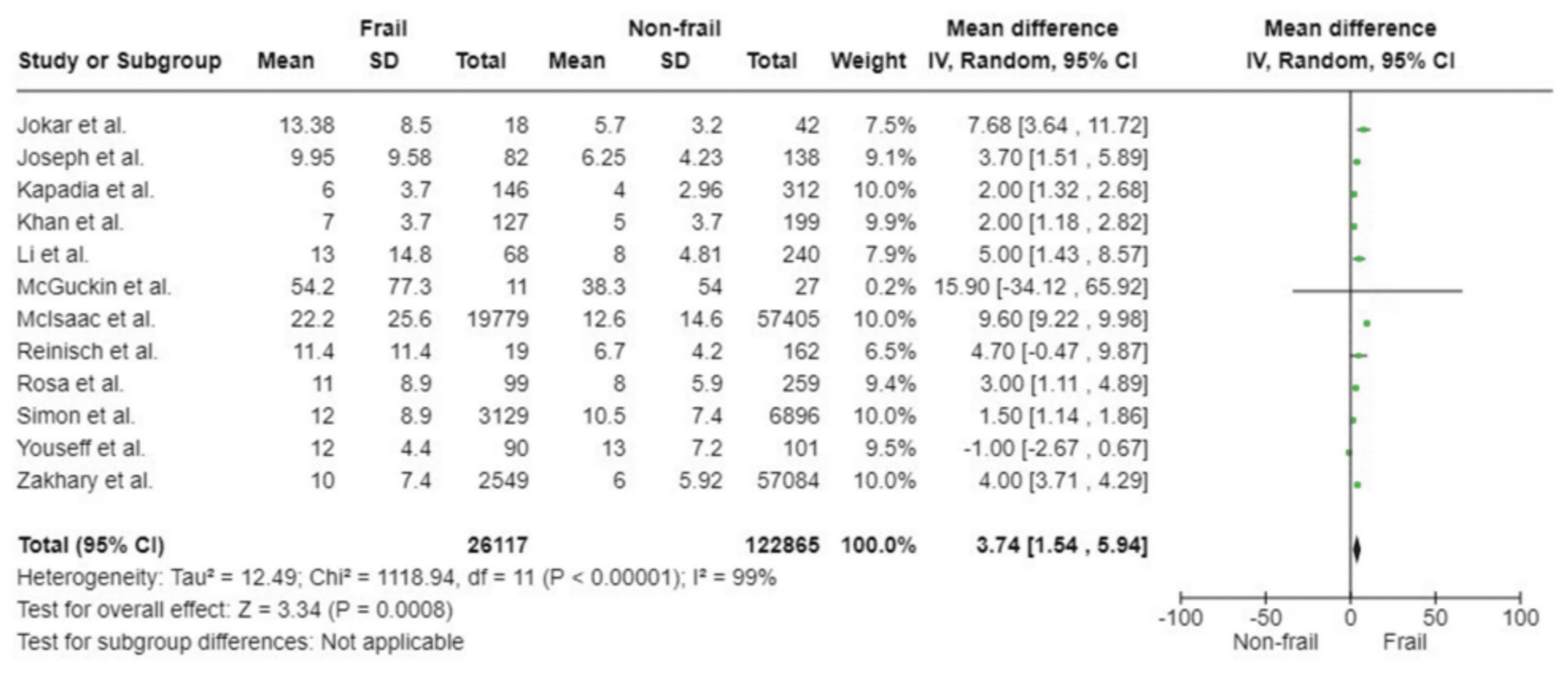
Forest plot of the association of frailty with duration of hospital stay References: [32,33,34,38,40,45,46,48,51,52]

Twelve studies covering a total of 148,982 participants analysed frailty and duration of hospital stay. Results of these studies were included in this pooled analysis. It showed that the frail group had a statistically significantly longer stay in hospital following emergency surgery compared to non-frail participants (WMD: 3.74; 95%CI: 1.54, 5.94; p<0.0008). However, the effect of heterogeneity cannot be ignored as considerable heterogeneity was noted amongst included studies with I^2^ of 99% (p<0.00001) (Figure 13).

Discharge Location

Studies included in this review assessed the discharge location of frail patients postoperatively. The pooled result of seven studies (958,903 participants) found that patients with frailty were less likely to be discharged to their usual home address compared to non-frail participants (OR: 0.28; 95%CI: 0.22, 0.38; p<0.00001). Whilst six studies (66,275 participants) assessed the association of frailty with discharge to a skilled nursing facility, another five studies (60,547 participants) evaluated the number of participants who were discharged to rehabilitation. The results of the pooled analysis showed that frail patients are twice as likely to be discharged to a skilled nursing facility (OR: 2.9; 95%CI: 2.00, 4.2; p<0.00001) or rehabilitation (OR: 2.47; 95%CI: 1.40, 4.35; p<0.002) following emergency surgery compared to the non-frail participants.

**Figure 14:**
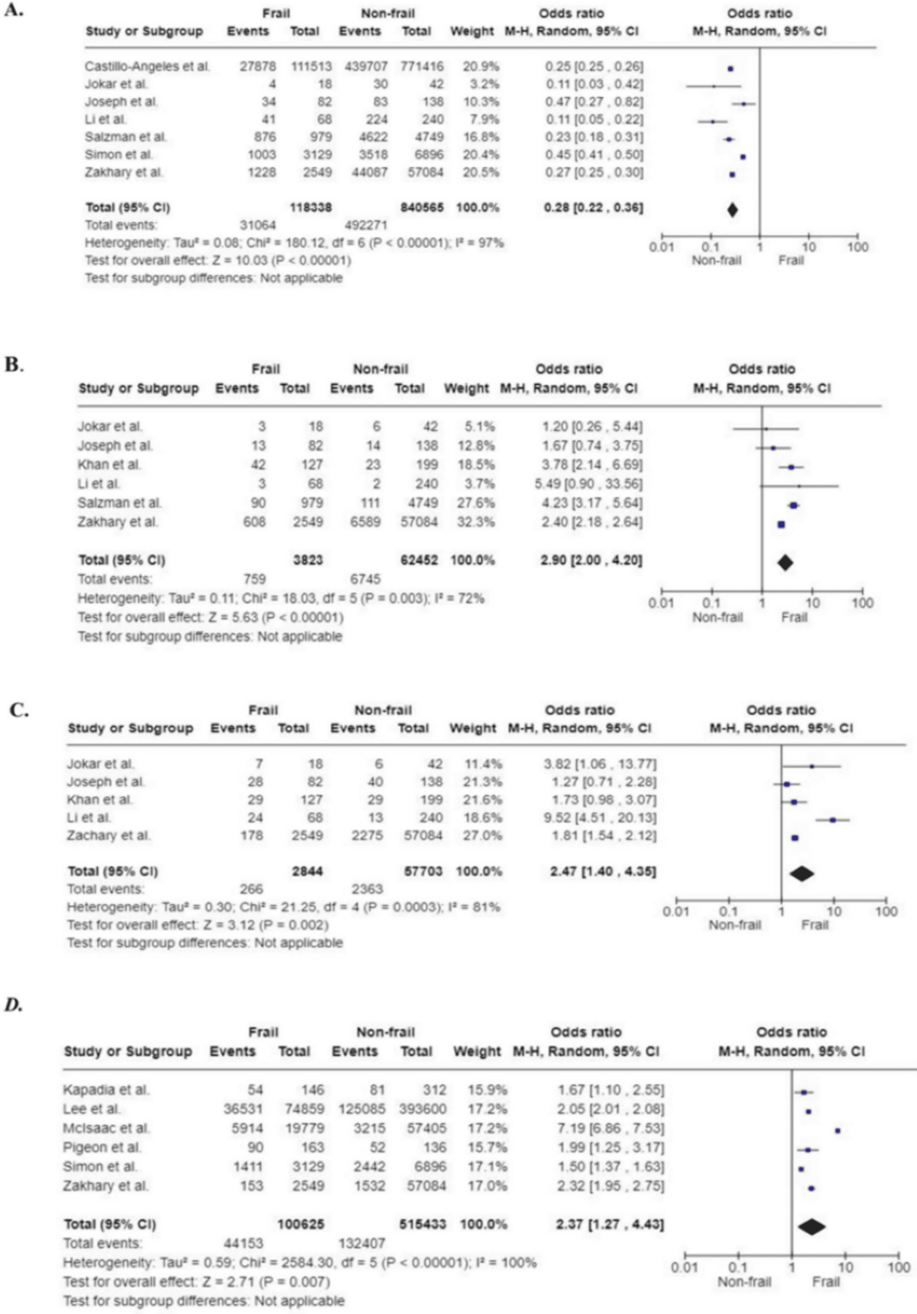
Forest plot of the association of frailty with discharge location. (A) Home (B) Skilled nursing facility (C) Rehabilitation (D) Non-home discharge. References: [27,32,33,34,38-40,47,48,52]

Six studies provided data in terms of frailty and non-home discharge. Patients living with frailty had a higher risk of being discharged to an alternative location other than home (OR: 2.37; 95%CI: 1.27, 4.43; p=0.007). For this outcome, the studies of Kapadia et al. and Simon et al. gave data on patients’ discharge to a nursing facility or rehabilitation collectively [34,48]. Lee et al. considered non-home location as that other than home or hospice [39]. McIsaac et al. provided data on institutional discharge, which was referred to as a long-term facility [42]. Similarly, the study by Pigeon et al. considered non-home discharge as discharge to an alternative address to the one on admission, suggesting that the patient requires rehabilitation, convalescence, or relocation upon discharge [44]. Conversely, for this outcome, the study of Zakhary et al. included discharge to hospice, separate acute care, and others. This gave rise to the significant heterogeneity of the pooled results (I^2^=100%; p<0.00001) (Figure 14) [52].

Re-admission

**Figure 15:**
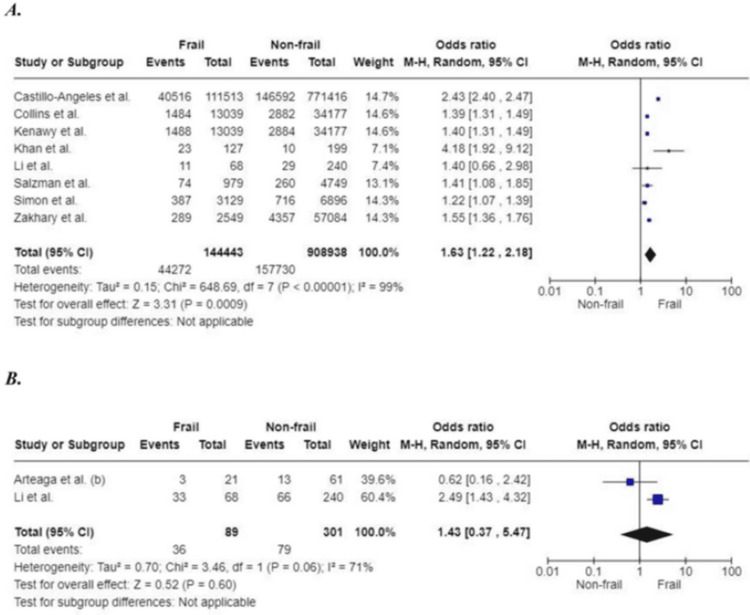
Forest plot of the association of frailty with re-admission at (A) 30 days and (B) six months References: [26,27,28,35,38,40,47,48,52]

Studies included in this review assessed patients’ readmission postoperatively at various time intervals (30 days, 6 months, 12 months, and 18 months). The pooled result of eight studies (1,053,381 participants) found that patients with frailty were more likely to be re-admitted within 30 days postoperatively (OR: 1.63; 95%CI: 1.22, 2.18; p=0.0009). The increased likelihood for frail patients to be readmitted was also noted at six months postoperatively (OR: 1.43; 95%CI: 0.37, 5.47; p=0.60). However, this finding, based on two studies and covering a total of 390 participants, was not found to be significant. The readmission rate at 12 months and 18 months was assessed in the studies of Vilches-Moraga et al. [50] and Arteaga et al. [26], respectively. As only one study was present at each time point, pooled analysis could not be performed. This study showed that patients living with frailty have a higher re-admission rate at 12 months (OR: 4.00; 95%CI: 1.74, 9.18; p=0.001) and 18 months (OR: 1.21; 95%CI: 0.46, 3.19; p=0.707) compared to non-frail participants (Figure 15).

Re-operation

**Figure 16:**
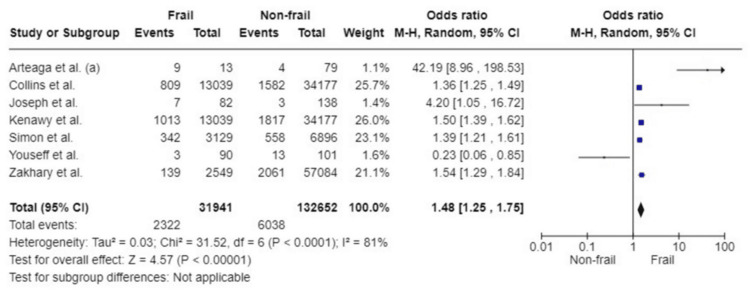
Forest plots of the association of frailty with re-operation References: [25,28,33,35,48,51,52]

Seven studies assessed the impact of frailty on reoperation. Pooled analysis was based on 31,941 participants in the frail group and 132,652 non-frail participants. Analysis of the results shows that patients living with frailty were significantly more likely to have a reoperation following an emergency surgical hospitalisation compared to non-frail patients (OR: 1.48; 95%CI: 1.25, 1.75; p<0.00001). Substantial heterogeneity was noted amongst the included studies (I^2^ =81%, p<0.0001) (Figure 16).

Subgroup Analysis

In this meta-analysis, the subgroup analysis was predefined, and the data were analysed based on different frailty scores, primarily CFS, m-FI, EGSFI, and CFI. Subgroup analysis was conducted for the main outcomes and when a sufficient number of studies were available. The findings from the subgroup analysis showed that these were consistent with the overall group effect.

Sensitivity Analysis

Sensitivity analyses were performed on the primary and secondary outcomes. These analyses assessed sources of heterogeneity based the sample size (studies with more than 100 participants in each arm), methodological quality (studies with a low risk of bias excluding moderate or high risk), study design (studies with a prospective research design, excluding retrospective studies), and surgery type (studies assessing frailty in patients undergoing emergency laparotomy only).

Results of the sensitivity analysis are displayed in Table 2.

**Table 2:** Results of the sensitivity analysis

Outcomes	No. of studies	No. of patients		Pooled results, WMD or OR (95%CI)	P-value	Heterogeneity	
		Frail	Non-frail			I^2 ^(%)	P-value
30-day mortality							
All studies included	20	222,707	1,309,437	2.83 (2.45, 3.27)	<0.00001	97	<0.00001
Sample size	13	22,373	1,308,822	2.66 (2.30, 3.09)	<0.00001	98	<0.00001
Quality	15	221,424	1,304,431	2.72 (2.35, 3.16)	<0.00001	98	<0.00001
Study design	8	3638	58,841	4.57 (3.20, 6.54)	<0.00001	55	0.03
Surgery type	4	75,299	394,699	2.99 (1.92, 4.68)	<0.00001	77	0.004
12-month mortality							
All studies included	5	131,630	560,045	1.97 (1.32, 2.93)	0.0008	100	<0.00001
Sample size	4	131,593	559,969	1.82 (1.20, 2.78)	0.005	100	<0.00001
Quality	5	131,630	560,045	1.97 (1.32, 2.93)	0.0008	100	<0.00001
Study design	-	-	-	-	-	-	-
Surgery type	3	109,788	497,700	2.04 (1.06, 3.93)	0.03	100	<0.00001
In hospital mortality							
All studies included	8	77,952	451,672	3.22 (1.91, 5.41)	<0.0001	96	<0.00001
Sample size	4	77,681	451,195	2.43 (1.42, 4.17)	0.001	98	<0.00001
Quality	7	77,880	451,634	3.00 (1.78, 5.08)	<0.0001	96	<0.00001
Study design	6	2994	57,813	3.55 (2.01, 6.26)	<0.0001	65	0.01
Surgery type	-	-	-	-	-	-	-
Postoperative complications							
All studies included	11	32,838	134,458	2.04 (1.90, 2.19)	<0.00001	65	0.002
Sample size	8	32,719	134,116	2.02 (1.89, 2.17)	<0.00001	71	0.001
Quality	10	32,819	134,296	2.04 (1.90, 2.19)	<0.00001	68	0.0009
Study design	7	3612	59,046	2.13 (1.84, 2.48)	<0.00001	33	0.18
Surgery type	-	-	-	-	-	-	-
ICU admission							
All studies included	4	20,015	57,867	2.15 (2.08, 2.22)	<0.00001	48	0.12
Sample size	2	19,906	57,604	2.15 (2.08, 2.22)	<0.00001	0	0.65
Quality	3	19,996	57,705	2.14 (2.08, 2.22)	<0.00001	35	0.21
Study design	-	-	-	-	-	-	-
Surgery type	-	-	-	-	-	-	-
ICU LOS							
All studies included	5	563	1438	1.12 (0.07, 2.16)	0.04	97	<0.00001
Sample size	3	463	1258	1.00 (-0.27, 2.27)	0.12	98	<0.00001
Quality	5	563	1438	1.12 (0.07, 2.16)	0.04	97	<0.00001
Study design	5	563	1438	1.12 (0.07, 2.16)	0.04	97	<0.00001
Surgery type	-	-	-	-	-	-	-
Duration of hospital stay							
All studies included	12	26,117	122,865	3.74 (1.54, 5.94)	0.0008	99	<0.00001
Sample size	5	25,730	121,896	3.83 (0.68, 6.97)	0.02	100	<0.00001
Quality	11	26,098	122,703	3.67 (1.40, 5.95)	0.002	99	<0.00001
Study design	6	2990	58,015	3.39 (2.12, 4.66)	<0.00001	89	<0.00001
Surgery type	-	-	-	-	-	-	-
Discharge location, home							
All studies included	7	118,338	840,565	0.28 (0.22, 0.36)	<0.00001	97	<0.00001
Sample size	4	118,170	840,145	0.29 (0.22, 0.39)	<0.00001	98	<0.00001
Quality	6	117,359	835,816	0.29 (0.22, 0.38)	<0.00001	97	<0.00001
Study design	4	2717	57,504	0.23 (0.13, 0.40)	<0.00001	75	0.007
Surgery type	-	-	-	-	-	-	-
Discharge location, skilled nursing facility							
All studies included	6	3823	62,452	2.90 (2.00, 4.20)	<0.00001	72	0.003
Sample size	4	3655	62,032	3.27 (2.09, 5.12)	<0.00001	87	0.0005
Quality	5	2844	57,703	2.49 (1.94, 3.19)	<0.00001	17	0.31
Study design	5	2844	57,703	2.49 (1.94, 3.19)	<0.00001	17	0.31
Surgery type	-	-	-	-	-	-	-
Discharge location, rehabilitation							
All studies included	5	2844	57,703	2.47 (1.40, 4.35)	0.002	81	0.0003
Sample size	2	2676	57,283	1.80 (1.55, 2.10)	<0.00001	0	0.89
Quality	5	2844	57,703	2.47 (1.40, 4.35)	0.002	81	0.0003
Study design	5	2844	57,703	2.47 (1.40, 4.35)	0.002	81	0.0003
Surgery type	-	-	-	-	-	-	-
Discharge location, non-home							
All studies included	6	100,625	515,433	2.37 (1.27, 4.43)	0.007	100	<0.00001
Sample size	6	100,625	515,433	2.37 (1.27, 4.43)	0.007	100	<0.00001
Quality	5	100,462	515,297	2.45 (1.24, 4.84)	0.01	100	<0.00001
Study design	2	2695	57,396	2.09 (1.55, 2.81)	<0.00001	50	0.16
Surgery type	-	-	-	-	-	-	-
Re-admission - 30-day							
All studies included	8	144,443	908,938	1.63 (1.22, 2.18)	<0.00001	99	0.0009
Sample size	7	144,375	908,698	1.65 (1.22, 2.24)	0.001	99	<0.00001
Quality	7	143,464	904,189	1.67 (1.22, 2.28)	0.001	99	<0.00001
Study design	3	2744	57,523	1.94 (1.11, 3.41)	0.02	68	0.05
Surgery type	-	-	-	-	-	-	-
Re-operation							
All studies included	7	31,941	132,652	1.48 (1.25, 1.75)	<0.00001	81	<0.0001
Sample size	4	31,756	132,334	1.44 (1.36, 1.52)	<0.00001	9	0.35
Quality	6	31,928	132,533	1.43 (1.29, 1.58)	<0.00001	58	0.04
Study design	3	2644	57,261	5.14 (0.82, 32.18)	0.08	89	<0.0001
Surgery type	-	-	-	-	-	-	-

Overall, the study findings are robust, as they consistently align with the original results despite variations in key variables such as sample size, study quality, and design, as well as the type of surgery. This consistency underscores the reliability of the association between frailty, adverse morbidity, and mortality outcomes in this patient population.

Publication Bias

**Figure 17:**
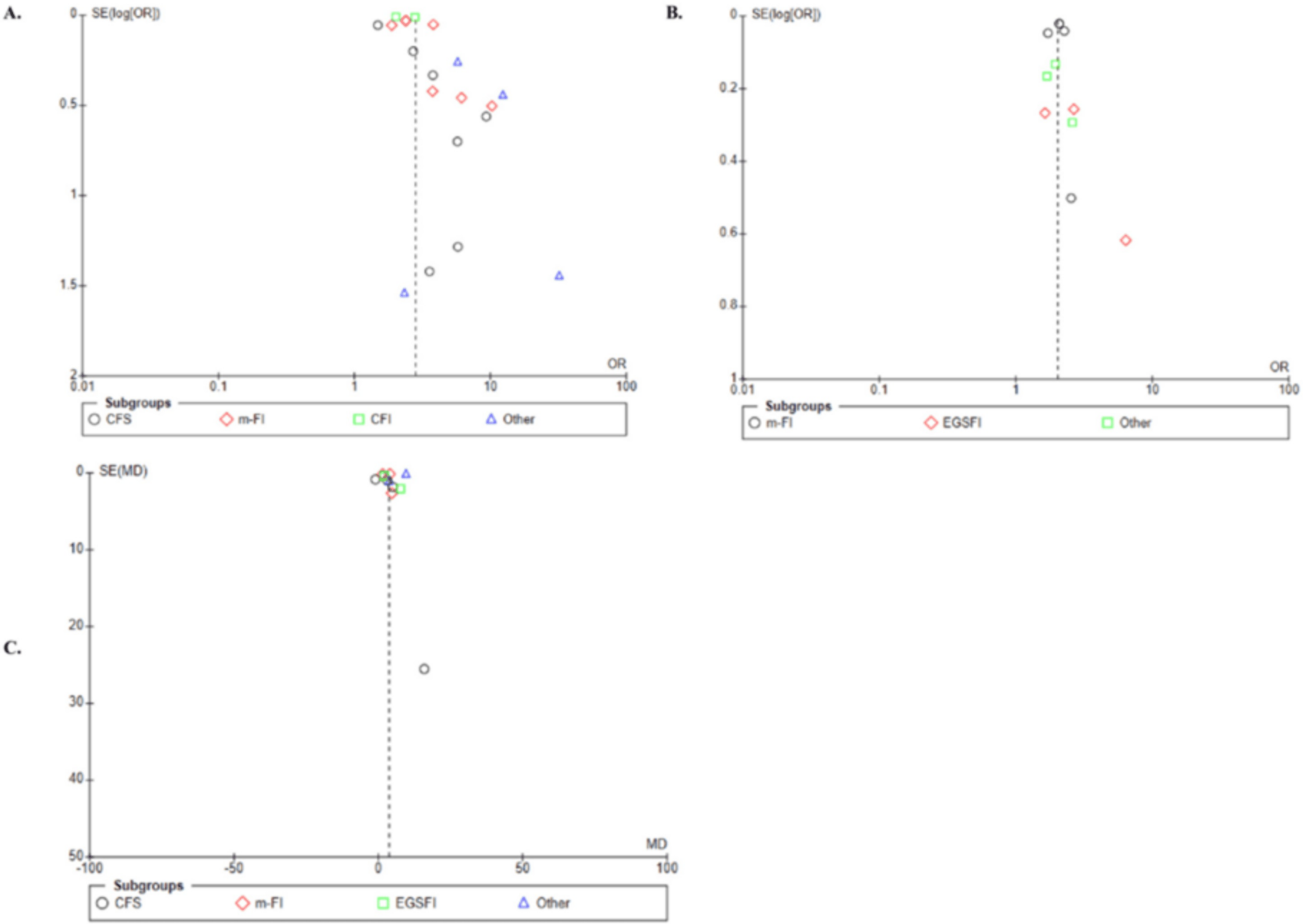
Funnel plots of publication bias (A) 30-day mortality (B) Postoperative complications (C) Duration of hospital stay.

Funnel plot analyses of the outcomes were conducted when more than 10 studies were included to enhance reliability. In this meta-analysis, publication bias was assessed through visual inspection of funnel plots. The funnel plots for postoperative complications and duration of hospital stay were symmetrically distributed, whereas the funnel plot for 30-day mortality showed asymmetry. This asymmetry suggests the potential presence of publication bias for this outcome, likely because studies with positive results are more frequently published. Consequently, this bias may affect the results of this meta-analysis (Figure 17).

Discussion

This meta-analysis investigated the relationship between preoperative frailty and postoperative outcomes in elderly patients undergoing emergency abdominal surgery. By analysing 31 studies that utilised various frailty instruments, the findings revealed a significant association between preoperative frailty and adverse postoperative health outcomes.

In this meta-analysis, the prevalence of frailty among geriatric patients undergoing emergency surgery was found to be 32.4%, with individual study estimates ranging from 4.3% to 76.7%. The prevalence of frailty among this population varies across different studies. For instance, a large study conducted in England found that approximately 43.7% of older adults (≥ 65 years) in primary care were classified as frail [55]. Another study involving critically ill older patients reported that 27.9% of those aged more than 65 years were classified as frail [56]. The meta-analysis by Gong et al. [57], incorporating 18 studies and 4479 elderly participants who underwent either elective or emergency surgery, found a frailty prevalence of 30%. This variation highlights the importance of context and setting in assessing frailty. The prevalence of frailty found in our meta-analysis was notably higher than the 10% reported by the British Geriatrics Society [58]. This discrepancy and wide variation in the prevalence of frailty may be attributed to the lack of standardisation in the definition and measurement of frailty, as various scales and concepts are employed across different studies. Additionally, differences in study populations and methodologies contribute to the wide range of reported prevalence rates for frailty. These variations underscore the need for a unified approach to define and assess frailty to obtain more consistent and comparable data. Nonetheless, irrespective of the actual prevalence figure, studies consistently indicate that a substantial portion of the elderly population is affected by frailty. While frailty can occur in younger age groups, the majority of individuals living with frailty are aged 65 years and older. This demographic constitutes a significant proportion of Western societies and is expected to grow. Consequently, there is projected growth in frail emergency surgical admissions, necessitating the need for surgeons to be able to manage an increasingly complex and frail patient population.

Mortality in the context of surgery is a critical metric that reflects both the risks associated with procedures and the outcomes of patient care. The mortality rate following emergency general surgery (EGS) ranges between 15% and 20%, depending on factors such as age and diagnosis [59]. The latest National Emergency Laparotomy Audit (NELA) states that in-hospital mortality is 9.2% [60]. The fifth NELA report also highlighted that frail patients over 70 years have a 30-day mortality rate of 23.4% versus 14.5% in non-frail peers [61]. Our meta-analysis further confirms this finding. In fact, our study found that frailty significantly increases the risk of mortality by at least twofold at 30 days, 180 days, and 12 months following emergency abdominal surgery. Frail patients were also found to have a significantly higher risk of mortality at 18 and 19 months, although these findings are based on a single study for each period. Patients living with frailty showed a threefold higher likelihood of in-hospital death. Additionally, frail patients exhibited a four-fold increase in 90-day postoperative mortality; however, this finding was not statistically significant, likely due to the small sample size and the inclusion of one study focusing solely on appendectomies, a low-risk procedure with low associated mortality. Our finding of the association between frailty and post-operative mortality aligns with previous meta-analyses, despite methodological differences. For instance, the study by Fehlmann et al. [62], which included adults aged 65 years and older undergoing various surgical procedures, and the study by Leiner et al. [63], which included patients aged 18 years and older admitted for general surgery, both demonstrated a two-fold increase in 30-day mortality rates.

Despite the established increased mortality rates in frail elderly patients and recommendations by international organisations such as the American and British Geriatrics Societies [58], NELA [60], and the WSES position paper [64], preoperative geriatric review is still not a routine practice in many countries, including the UK. These services integrate geriatric team input into the surgical pathway, aiming to enhance perioperative care for older patients. Most evidence for these types of services comes from elective surgery studies, with few studies evaluating the EGS cohort, despite the high prevalence of frailty in this group. Barriers to implementing preoperative frailty assessment include the lack of time to administer and score lengthy frailty assessments in both emergency settings and busy preoperative clinics, making such scoring unfeasible. The literature features over 50 tools for frailty assessment, varying by intended use, practitioner, and target population. Selecting the most appropriate tool for EGS remains a challenge, and it is evident that some tools used in studies are impractical for everyday use [65]. In our meta-analysis, most of the included studies used the CFS score. The latter frailty score has been shown to demonstrate both accuracy in predicting mortality and feasibility for clinical practice. Another obstacle to implementing preoperative geriatric review is the limited availability of geriatricians and advanced care practitioners, especially outside regular working hours. Unlike elective surgeries, emergency surgeries are unplanned and can occur at any time, exacerbating this challenge and limiting frailty assessment [51].

Frailty not only affects mortality rates but is also associated with increased complications and institutionalisation, underscoring the lasting physical and cognitive disabilities of frail patients after surgery. Our meta-analysis indicates that frail patients have a two-fold increased risk of postoperative complications, including severe complications graded as Clavien-Dindo III or higher. Research indicates that frail patients possess diminished physical reserves, thereby limiting their ability to recover optimally from surgical procedures. Moreover, these individuals often suffer from multiple comorbidities, increasing the risk of post-operative complications like myocardial infarction and respiratory failure. Despite heterogeneity amongst the studies, we found a statistically significant association between frailty and postoperative complications across various body systems, including respiratory, cardiac, urinary, skin, and renal systems. Furthermore, studies have demonstrated that frailty correlates with heightened inflammation, potentially due to elevated levels of acute-phase reactants and coagulation factors such as CRP, factor VIII, and fibrinogen. This inflammatory response may increase following major surgery, contributing significantly to complications, particularly those related to impaired wound healing [66,67]. Higher complication rates of frail patients contribute to increased ICU admissions, longer hospital stays, and a greater likelihood of discharge to non-home settings. These findings in our study were found to be statistically significant and are consistent with other international studies.

The rate of readmission following surgery reflects the quality of care provided during the initial hospitalisation and discharge process. Similarly, a high re-operation rate typically indicates that complications or issues related to the initial surgery have arisen, necessitating additional surgical intervention. Our meta-analysis revealed significantly higher rates of reoperation and 30-day readmission following emergency abdominal surgery among frail patients compared to their non-frail counterparts. The higher reoperation and re-admission rates in frail patients are likely to be attributable to their higher complication rates.

Previous studies have often highlighted age and comorbidities as key predictors of outcomes in elderly patients undergoing emergency abdominal surgery [68-71]. However, our focused analysis on the geriatric population reveals that frailty, evaluated across different frailty scores, is a critical indicator of adverse postoperative outcomes, including mortality. Our meta-analysis has been strengthened by the use of subgroup and sensitivity analyses. These efforts have consistently reinforced the primary findings, underscoring the robustness and reliability of our conclusions.

Implications to Practice

The link between frailty and postoperative risks and morbidity and mortality carries clinical implications. Patients and families must have realistic expectations, as frail patients face increased complications and may require rehabilitation or institutionalisation. Frail patients may opt for non-surgical alternatives, whilst those with lower frailty scores may choose surgery to improve quality of life. Quick, accurate frailty assessments can guide further geriatric evaluations and targeted treatments.

For surgeons, our meta-analysis underscores the importance of carefully considering all treatment options for very frail elderly patients. Identifying clinically vulnerable and frail individuals is crucial for managing expectations and obtaining informed consent prior to emergency surgeries. This meta-analysis stresses the need for an efficient multidisciplinary team, including surgeons, geriatricians, physiotherapists, specialist nurses, rehabilitation services, and dietitians from the moment a patient is assessed for an emergency surgery. Early interventions include nutrition counselling and a protein-rich diet, as well as implementing physiotherapy and pulmonary rehabilitation to reduce postoperative complications and enhance support for home discharge. As frail patients have increased postoperative risks and FTR, increased vigilance and additional assessments, such as laboratory tests or imaging during the postoperative period, can help mitigate risks. Ensuring adequate perioperative support is essential for better recovery.

For healthcare administrators, these findings inform resource allocation, postoperative planning, and optimal bed management to better support frail geriatric patients after emergency surgery.

Strengths and Limitations

To our knowledge, this meta-analysis offers the most comprehensive review of literature with the most rigorous methodology to date, assessing the association between frailty and EGS in elderly patients. It incorporates a wide variety of validated frailty scores and includes the largest sample size available in the current literature. This study successfully fulfilled its aims and answered the research questions with statistically significant findings. The consistency of our results with previous meta-analyses further confirms the reliability.

This meta-analysis has several limitations. All included studies were observational, some with small sample sizes and methodological concerns, potentially affecting the pooled results. Confounding biases may also weaken the findings. The retrospective design of some studies means data quality depends on medical record accuracy. The study covered various EGS, from hernias to laparotomies, performed by different teams, introducing variability in surgical skill, technique, and outcomes. Significant heterogeneity in clinical outcomes necessitates cautious interpretation. Studies involving younger patients, conservative treatment, or different surgeries (e.g., orthopaedics) were excluded unless stratified data were provided. Additionally, studies presenting only odds ratios were excluded. Kenawy et al. [35] and Collins et al. [28] used the same database for the same period, yielding similar outcomes. Selection bias is another concern, as included studies likely only analysed frail patients deemed fit for surgery, excluding those not operated on. However, this reflects real-world decision-making. Finally, our meta-analysis categorised data into frail and non-frail groups, with participants classified as pre-frail included in the non-frail group, which could potentially influence the pooled results.

## Conclusions

This meta-analysis shows that frailty affects one-third of older adults (≥65 years) undergoing emergency abdominal surgery and is a strong predictor of poor postoperative outcomes, including higher morbidity, mortality, prolonged hospital stay, and increased readmission and re-operation rates. These findings support the routine use of preoperative multidisciplinary assessments. Future research should integrate frailty scores with other prognostic risk factors to develop robust tools and examine frailty's impact on long-term functional outcomes.
